# Numerical Analysis and Its Laboratory Verification in Bending Test of Glue Laminated Timber Pre-Cracked Beam

**DOI:** 10.3390/ma12060955

**Published:** 2019-03-22

**Authors:** Bartosz Kawecki, Jerzy Podgórski

**Affiliations:** Lublin University of Technology, Faculty of Civil Engineering and Architecture, ul. Nadbystrzycka 40, 20-618 Lublin, Poland; j.podgorski@pollub.pl

**Keywords:** FEA, glue laminated timber, GLT beam, GLT damage, laboratory testing

## Abstract

The paper describes an approach to model glue laminated timber (GLT) made of pinewood (softwood), taking into account damage processes in timber and adhesive layers. An example of a pre-cracked beam has been presented. Obtaining necessary material properties has been supported by several finite elements models (FEM), laboratory tests and a wide literature survey. The authors have taken into consideration an orthotropic material with damage for timber lamellas and isotropic traction separation law for bonding layers. Both plane stress CPS8R and COH2D4 finite elements from the Simulia ABAQUS system or mesh density, used in the numerical analyses, have been carefully chosen and tested. The GLT beam model has been verified on the basis of several values, using two types of testing techniques—MTS 809 machine measurements and digital image correlation (DIC). Additional results revision has been provided by measuring a crack shear displacement (CSD), using the original laboratory test setup by the authors’ idea. Finally, some conclusions and introductory recommendations for modelling a glue laminated timber in a plane stress have been formulated. The paper is a part of a wider research on timber-polymer composites, which is still under development.

## 1. Introduction

**Figure 1:**
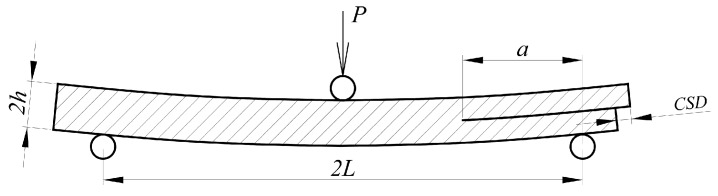
End notched flexure (ENF) test specimen and definition of *a*, *L*, *h*, *CSD*.

Authors of the papers [1,2,3,4], proposed the methods for calculating an energy release rate in mode II, on the basis of the end notched flexure test (ENF), presented in Figure 1. In the papers [1] and [2], the idea of crack shear displacement (CSD) measurement was introduced. The formulas derived for fracture energy calculation were based on the elementary elastic beam theory. The proposed model defined the *a/L* ratio—a crack length to a half span of the beam, but it did not define the *L/h* ratio—a half span to a half height of the beam. On the basis of this observation, there was an attempt made to adopt the method to beams of real dimensions, used in construction. An original laboratory test setup for measuring the CSD value in the pre-cracked beam was created. Four pre-cracked beam samples with the *a/L* = 0.7 ratio, specified as the value for stable crack propagation, were prepared. The meaning of the *a*, *L*, *h*, and *CSD* are given in Figure 1.

Problems occurred just at the beginning of the test. It turned out that the timber was crushing near the machine rolls. Crushing deformations almost doubled the beam deflection. Such large deformations caused all of the proposed formulas to be useless in this particular case.

Taking advantage of this experience, the authors decided to examine processes occurring in the tested samples using detailed finite element method with advanced material properties. A plane stress, elastic orthotropic material [5,6], with Hashin damage initiation criteria [7,8], and linear softening law [9,10,11], was proposed for timber. Hashin criteria were generally developed for carbon-epoxy composites with unidirectional fibers, which have been explained in detail in the cited publications. On the basis of the statement that timber fibers in a glue laminated timber (GLT) were precisely positioned, there might be assumed similarities to unidirectional carbon-epoxy composites. Approaches to model the timber using Hashin criteria and linear fracture mechanics were done in the several publications [12,13,14]. The proposed damage model gave satisfactory results in all of the cases. For modelling the glue surfaces, a cohesive zone theory was used. Recommendations for the parameter’s selection were taken from the papers [15,16,17].

There was proposed a laboratory verification of the finite elements analysis (FEA) results in several steps. The first one was the measurement of the *P*(*δ*) relation—a force value to a vertical displacement of the rolls. Second, the horizontal displacement, called crack shear displacement (CSD), was compared. Additionally, there a simple approach was made to obtain the mid-span deflection and the crushing deformations near the loading and supporting points, using a digital image correlation (DIC) method. All tests were performed on GLT elements made of the same batch of pinewood and kept in laboratory conditions for three months before testing. The aim was to ensure the same humidity conditions for all of the tested beams. Defects as knots and finger connections were carefully omitted in a cutting process to provide as homogenous a material, as possible. The paper is a part of the timber-polymer composites numerical modelling research.

## 2. Timber and Adhesive Properties

To obtain elastic parameters for the timber, compression tests were performed. The adhesive properties were determined from a double lap shear test. Methods of testing and selecting elastic properties of the glue laminated timber and damage parameters for the adhesive layer were precisely described in the authors’ previous publication [18]. Timber strengths were assumed as for the GL24h class, based on the elastic parameter’s values. The timber strengths were taken from [19] and fracture energies from [20]. The influence of a viscosity coefficient in the numerical analyses, using ABAQUS, was analyzed in [21]. An appropriate value, providing good quality results was chosen for the computations. All of the properties are presented in Table 1.

## 3. Laboratory Tests on the Pre-Cracked Beams

**Figure 2:**
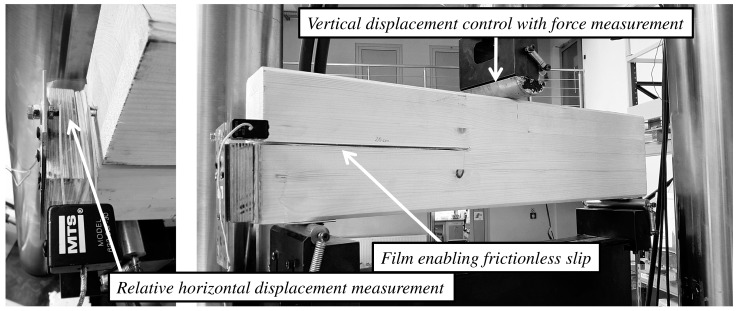
Pre-cracked beam—laboratory setup.

The laboratory experiments included a three point bending test on the 4 samples with a pre-crack in the middle of the height (Figure 2). Not to duplicate the figures, precise dimensions are given in Figure 8, in the “finite elements models (FEM) model assumptions” chapter. The tests were done on MTS 809 machine (MTS Systems Corporation, Eden Prairie, MN, USA). A stable vertical displacement with a force measurement was provided. Additionally, the relative horizontal displacement (CSD) was measured, using the authors’ setup with a gap gauge. The laboratory test setup are presented in Figure 2.

**Figure 3:**
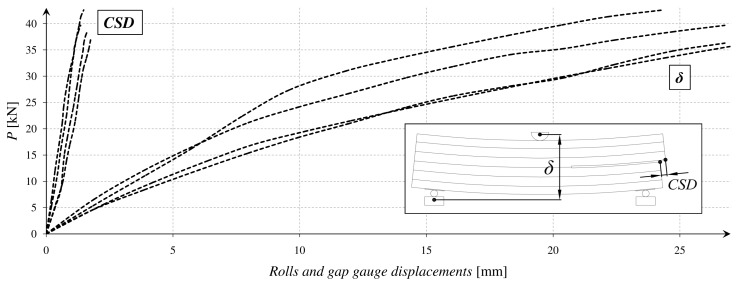
Experimental *P*(*δ*) and *P(CSD)* relations.

On the basis of the tests, a diagram for relations *P*(*δ*) and *P(CSD)* was created. It is presented in Figure 3.

**Figure 4:**
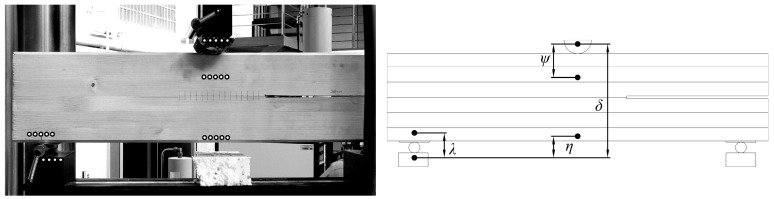
Digital image correlation (DIC) method and *δ*, *λ*, *ψ*, *η* parameters determination.

**Figure 5:**
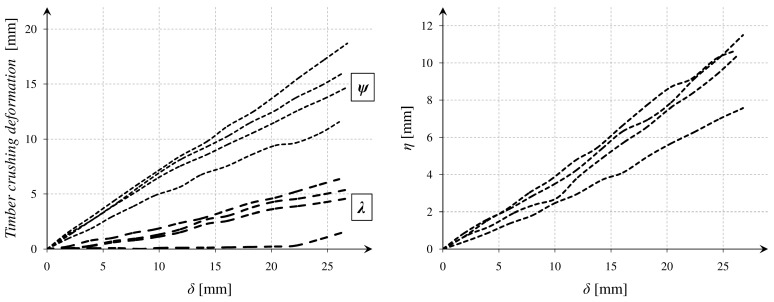
Experimental *ψ(δ)* and *η(δ)* relations.

In parallel, there were performed simple measurements with DIC method. Special markers were glued to the beam, and a movie was recorded with the Panasonic 4K camera (Panasonic Corporation, Osaka, Japan). Next, frames were processed in a GOM Correlate software (2016, GOM GmbH, Braunschweig, Germany). After executing all of the operations, it was possible to read out the vertical displacements of the points. The results were correlated with those obtained from the testing machine, based on a relative vertical displacement of the rolls *(δ)*, which was measured in both cases. The DIC method enabled to capture relations between the several important parameters as *λ*, *ψ*, and *η*, which are marked in Figure 4. The relations *ψ(δ), λ(δ)*, and *η(δ)* are presented in Figure 5.

The parameter *η* might be defined as a beam deflection in the middle of the span. The parameter *ψ* described a deformation which was caused by the timber crushing near the top machine roll. The parameter *λ* was similar to the previous one but described a deformation near the bottom machine roll. The influence of crushing was especially significant in the case of the tested softwood, where the damage processes in a transversal direction started very early.

## 4. FEM Model Assumptions

**Figure 6:**
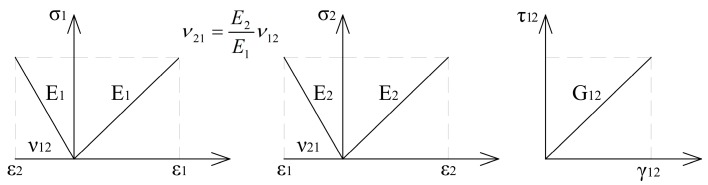
Constitutive law for an elastic orthotropic material model representing timber.

To model the behavior of the GLT beam in a plane stress, the authors proposed to use Simulia ABAQUS software (2019, Dassault Systemes, Paris, France). A constitutive law for a plane stress orthotropic timber material (Equation (1) and Figure 6) was defined as in [18] and [22]:

(1) $$\left[\begin{array}{c} \varepsilon_{11}\\ \varepsilon_{22}\\ \gamma_{12} \end{array}\right]=\left[\begin{array}{ccc} \;\frac{1}{E_{1}} & -\frac{\nu_{12}}{E_{1}} & 0\\ -\frac{\nu_{21}}{E_{2}} & \;\frac{1}{E_{2}} & 0\\ 0 & 0 & \frac{1}{G_{12}} \end{array}\right]\left[\begin{array}{c} \sigma_{11}\\ \sigma_{22}\\ \tau_{12} \end{array}\right]$$

Hashin damage initiation criteria [7,8], are described below and given by the formulas (2–5). The particular strengths of the timber *f_t,1_*, *f_c__,1_, f_t,2_, f_c__,2_*, and *f_v__,12_* are defined in Table 1 in Chapter 2. The parameter determining shear participation in the fibers rupture if the tension was set *α* = 1 as defined in [8]. The names of the failure modes are adopted from the paper [9].

The timber’s fibers rupture in tension is described with equation:

(2) $${\left(\frac{\sigma_{11}}{f_{t,1}}\right)}^{2}+{\left(\frac{\tau_{12}}{f_{v,12}}\right)}^{2}=1\;for\;\sigma_{11}\geq 0$$

The timber’s fibers buckling and kinking in compression is expressed by formula:

(3) $${\left(\frac{\sigma_{11}}{f_{c,1}}\right)}^{2}=1\;for\;\sigma_{11}<0$$

The timber’s matrix cracking under transverse tension and shearing is described with equation:

(4) $${\left(\frac{\sigma_{22}}{f_{t,2}}\right)}^{2}+{\left(\frac{\tau_{12}}{f_{v,12}}\right)}^{2}=1\;for\;\sigma_{22}>0$$

The timber’s matrix crushing under transverse compression and shearing is expressed by formula:

(5) $$\left[{\left(\frac{f_{c,2}}{2f_{v,12}}\right)}^{2}-1\right]\left(\frac{\sigma_{22}}{f_{c,2}}\right)+{\left(\frac{\sigma_{22}}{2f_{v,12}}\right)}^{2}+{\left(\frac{\tau_{12}}{f_{v,12}}\right)}^{2}=1\;for\;\sigma_{22}<0$$

**Figure 7:**
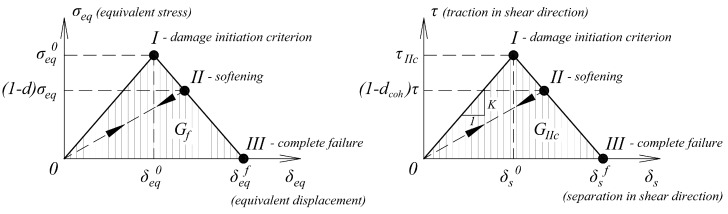
Graphical representation of bi-linear softening law for timber and adhesive material.

The response of the timber damaged material was calculated from the general formulas (Equations (6)–(8)), where *d_f_* and *d_m_* were the damage rates for the fibers and the matrix. Subscripts *t* or *c*, after comma, were consecutively tension or compression. The denotation *d_s_* describes the damage parameter in shearing, which was calculated from the variables mentioned previously. After reaching the damage initiation criterion, the linear softening of the material progressed. A graphical representation of the law is presented in Figure 7. A more detailed study about the material model was carried out in [23].

(6) $$\sigma =\frac{1}{D}\left[\begin{array}{ccc} \left(1-d_{f}\right)E_{1} & \left(1-d_{f}\right)\left(1-d_{m}\right)\nu_{21}E_{1} & 0\\ \left(1-d_{f}\right)\left(1-d_{m}\right)\nu_{12}E_{2} & \left(1-d_{m}\right)E_{2} & 0\\ 0 & 0 & \left(1-d_{s}\right)DG_{12} \end{array}\right]\varepsilon$$

(7) $$d_{s}=1-\left(1-d_{f,t}\right)\left(1-d_{f,c}\right)\left(1-d_{m,t}\right)\left(1-d_{m,c}\right)$$

(8) $$D=1-\left(1-d_{f}\right)\left(1-d_{m}\right)\nu_{12}\nu_{21}$$

Cohesive layer damage initiation criterion (maximum shear stress) is given by the formula:

(9) $$\frac{\tau }{\tau_{IIc}}=1$$

The response of the adhesive damaged material was calculated from the general formula (Equation (10)). The material in the undamaged state was assumed to be elastic isotropic. The penalty stiffness *K* was calculated based on the stiffness of the bonded elements. Precise principals for the penalty stiffness selection are presented in several papers [15,16,17] and analyzed by the authors of [22]. Graphical representation of the traction-separation law for cohesive elements [24] is given in Figure 7.

(10) $$\tau =\left(1-d_{coh}\right)K\delta_{s}$$

**Figure 8:**
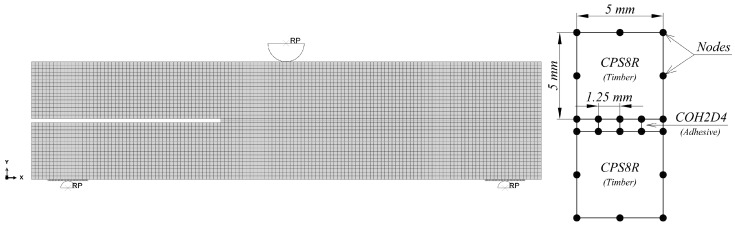
Meshed model and elements description.

The timber was modelled with CPS8R elements, whereas the adhesive layer with COH2D4 elements. The model was meshed uniformly with the quadrilateral 8-node elements of 5 × 5 mm dimensions. The mesh 2.5 × 2.5 mm gave the same results but much slower computations. Such a meshing technique was assumed, because the ABAQUS documentation [23] recommends the use of the quadrilateral elements for the damage problems. Additionally, element’ quality was studied in [25]. The cohesive element length was defined as 1.25 mm [18]. This resulted in four COH2D4 elements per one CPS8R element (Figure 8). The nodes compatibility was not required, because a “TIE” constraint between the connected parts was applied [23].

**Figure 9:**
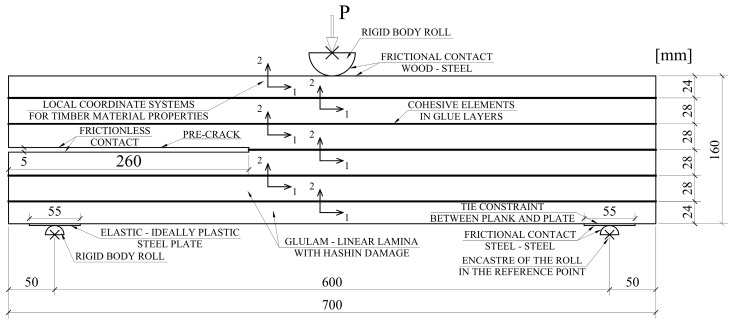
Finite elements method (FEM) model detailed description.

The out-of-plane thickness was assumed as 80 mm, as it was in reality. The same assumptions were made in the previous research on shear delamination in glulam elements [18]. Timber planks were suitably oriented to ensure fibers set in the first axis direction, which was necessary for an orthotropic material. Frictional contacts, steel–steel (*μ* = 0.15) and wood–steel (*μ* = 0.5), were added to capture the interactions between the machine rolls and the sample. The points with a frictional contact were additionally treated as boundary conditions. The film enabling slip, in case of mutual beams contact in the pre-crack, was modelled as a frictionless contact. The class of a steel plate, situated at the bottom face, was S185, as the producer declared. The height of the plate was 2 mm. It was modelled as an elastic–perfectly plastic. Elements deletion was not allowed to avoid convergence problems in the calculations. A detailed model description, with the boundary conditions and other assumptions, are given in Figure 9.

## 5. Results and Discussion

**Figure 10:**
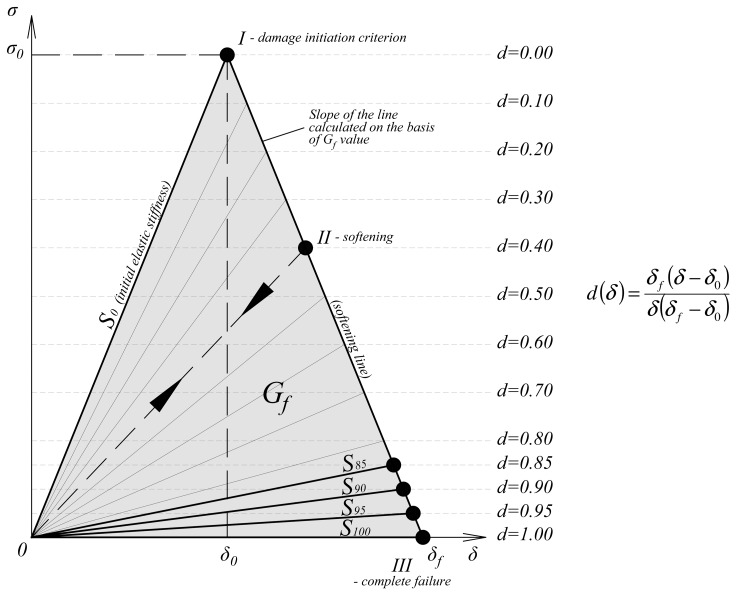
Damage rate parameter *d*—explanation.

The aim of the paper was an attempt to describe failure processes occurring in a glue laminated timber made of softwood. The chosen method was a FEA with damage taken into consideration. Generally, a non-linear finite elements analysis is affected by uncertainties [26]. Therefore, the correlation between the laboratory tests and the numerical modelling was necessary to be obtained. The model was fitted to the experiments by the modification of the damage rate parameter *d* for timber material. Simulia ABAQUS software enabled that modification for the whole material model [23]. Unfortunately, it was impossible to differentiate the variables *d_f_* and *d_m_*. Both of them were set at the same level. In future work, the authors plan to create a similar material model with different values of *d_f_* and *d_m_* parameters. An explanation of the influence of changing parameter *d* is given in Figure 10 and in the description presented below.

When the damage initiation criterion is not met—the damage variable is equal to *d* = 0.00. It means that elements remain with the initial linear elastic stiffness *S_0_*, defined at the beginning of the analysis. After reaching a damage initiation criterion, the damage variable grows continuously from *d* = 0.00 to *d* = 1.00, as a default option. It means that the stiffness of the material is reduced until zero stiffness *S_100_* occurs.

Changing the damage variable *d* to a different value, for example, *d* = 0.90, results in a continuous stiffness reduction of the material to the *S_90_*. After reaching the *d* = 0.90 value, the stiffness remains linear elastic, at the reduced level of *S_90_*, until the end of the analysis. The principle is the same for all of the randomly selected damage parameters from the range of 0.00 ÷ 1.00.

The damage variable for the cohesive layers was set at the default level equal to *d* = 1.00, with elements deletion turned off, to obtain better convergence of the calculations. It was determined by the previous authors’ numerical analysis and the laboratory experiments, presented in paper [18].

For the timber, four values of the damage parameter: *d* = 1.00 (default option) were tested, *d* = 0.95, *d* = 0.90 and *d* = 0.85. The first one (*d* = 1.00) was omitted in the results presentation, because of the very low consistency with the laboratory tests. The best fitting to the average experimental curve *P*(*δ*) was obtained for the value equal to *d* = 0.90. The fitting error is given in Table 2.

**Figure 11:**
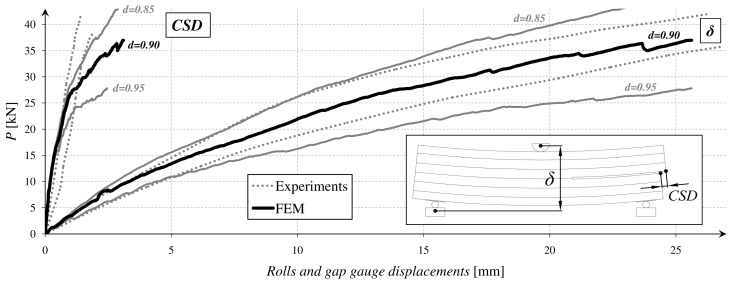
Correlation of the values measured by machine and gap gauge.

**Figure 12:**
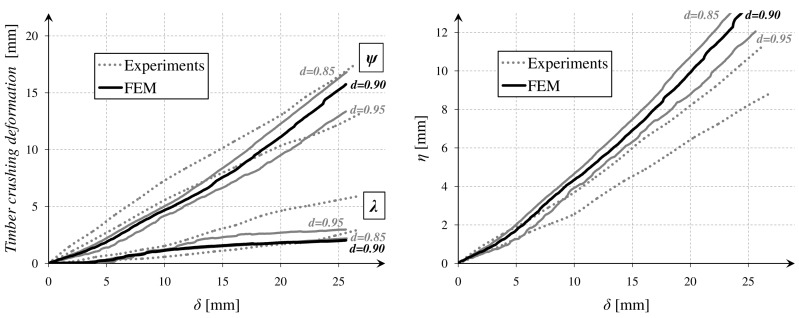
Correlation of the values measured by DIC method.

After the calibration of the FEM model, other parameters were compared—*P(CSD)*, *ψ(δ)*, *λ(δ)*, and *η(δ)*. It enabled to draw some general conclusions about a material model application and its future improvement. All of the correlations were presented in the diagrams in Figure 11 and Figure 12.

The dotted lines showed the envelopes of the results from the laboratory tests. The envelope values were calculated on the basis of the fundamental statistical formulas (Equations (11) and (12)). The *V*(*δ*) defined the variables from the tests (consecutively *P*(*δ*), *P*(*CSD*), *ψ*(*δ*), *λ*(*δ*)**, and *η*(*δ*)) and *n* is the number of the samples.

(11) $$V{\left(\delta \right)}_{avg}=\;\frac{\sum_{i=1}^{n}V{\left(\delta \right)}_{i}}{n}$$

(12) $$V{\left(\delta \right)}_{\sigma_{x}}=\;\frac{\sqrt{\sum_{i=1}^{n}{\left(V{\left(\delta \right)}_{i}-V{\left(\delta \right)}_{avg}\right)}^{2}}}{n}$$

All results from numerical analyses seemed to be satisfactory. The crack shear displacement was small, and the differences might be caused by the imperfections in mounting a gap gauge in laboratory conditions. Overestimation of the middle-span deflection might be caused by an equal limitation of the damage rate parameter. As was mentioned before, another study should be made to distinguish damage rate for both the matrix (*d_m_*) and fibers (*d_f_*). The timber crushing deformations were similar to those obtained from the laboratory tests. The research confirmed the statement, that proposed material models, after some modifications and additional experimental calibrations may be correctly used to model a glue laminated timber in a plane stress assumption.

**Figure 13:**
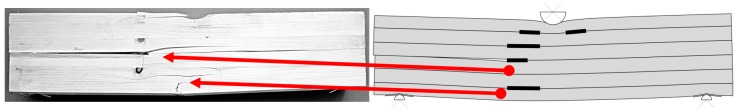
Adhesive delamination identification.

**Figure 14:**
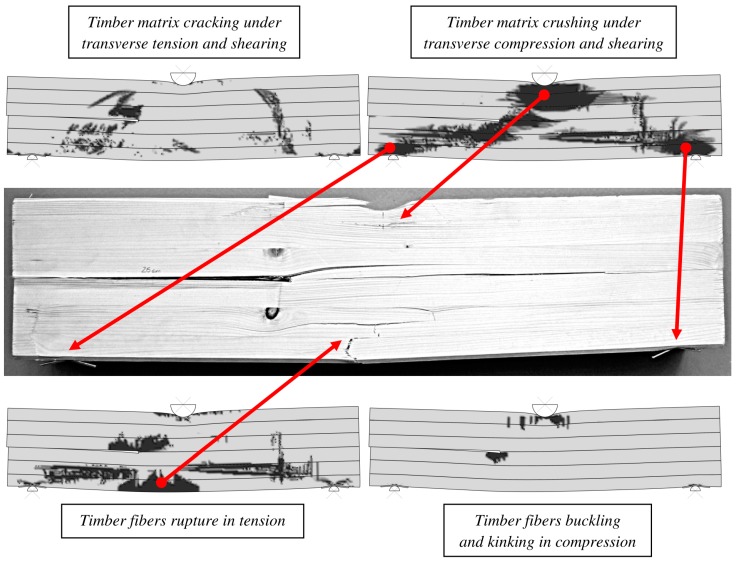
Timber damage identification.

The next step of the analysis was a comparison of the damage patterns between the calibrated finite element model and the experimental results. Bitmaps with the damaged areas for the different damage criteria were prepared, defined in the material model (black color in Figure 13 and Figure 14).

The full delamination of the bonding surface did not appear in the numerical model, but there was observed an advanced softening of the glue layers, marked in Figure 13.

The most visible damaged regions in the timber were also identified in the laboratory samples (Figure 14). The FEM model enabled to capture the crushing behavior of a softwood near the top and the bottom machine rolls. It was well described by the matrix crushing under transverse compression and shearing. Additionally, the fiber rupture locations in tension was suitably reproduced on the sample, comparing to the laboratory experiments.

## 6. Conclusions

Concluding, the series of numerical analyses and laboratory tests confirmed the possibility of modelling glue laminated timber, using Hashin damage model for wood and traction-separation law for adhesive. Both progressive damage models were accessible in Simulia ABAQUS software. Timber anisotropic material was brought to the orthotropic one and the results which occurred were satisfactory.

It was proved that the model can reproduce the measured test responses. Despite this fact, the type and amount of control parameters require more accurate study in simpler experimental tests and numerical analyses. This model is primarily functional only when associated with validating experimental tests. After some future modifications and calibrations, similar model assumptions may be used to observe and describe damage processes in glue laminated timber or timber-polymer composites.

The model is an introductory work on damage and delamination in timber-based composites. At this stage, because of many variables, it is not accurate enough to be used for the final prediction of the structural behavior of GLT beams. The work is the part of a wider research on numerical modelling of timber-polymer composites, which is still under development.

## Figures and Tables

**Table 1:** Material parameters tested, selected, and used in numerical analyses.

Description	Parameter	Value
timber longitudinal elastic modulus	*E_1_*	11.65 GPa
timber transversal elastic modulus	*E_2_*	0.31 GPa
timber shear modulus	*G_12_*	0.69 GPa
timber Poisson coefficient	*ν_12_*	0.35
timber longitudinal tensile strength	*f_t,1_*	16.5 MPa
timber transversal tensile strength	*f_t,2_*	0.4 MPa
timber longitudinal compression strength	*f_c,1_*	24.0 MPa
timber transversal compression strength	*f_c,2_*	2.7 MPa
timber shear strength	*f_v,12_*	2.7 MPa
timber energy release rate for longitudinal direction	*G_f1_*	920 J/m^2^
timber energy release rate for transversal direction	*G_f2_*	250 J/m^2^
adhesive penalty stiffness	*K*	12.95 GPa/mm
adhesive damage initiation shear stress	*τ_IIc_*	5.05 MPa
adhesive energy release rate	*G_IIc_*	807 J/m^2^
viscosity coefficient	*γ*	0.0001

**Table 2:** *P*(*δ*) curve fitting error.

*δ* [mm]	*P*(*δ*) [kN] (*Experiments*)	*P*(*δ*) [kN] *d* = 0.85	*P*(*δ*) [kN] *d* = 0.90	*P*(*δ*) [kN] *d* = 0.95
5	12.57	15.61 (24.2%)	**13.44 (6.9%)**	10.95 (12.9%)
10	23.67	26.25 (10.9%)	**21.96 (7.2%)**	16.25 (31.3%)
15	28.82	33.95 (17.8%)	**28.35 (1.6%)**	21.57 (25.2%)
20	32.77	39.81 (21.5%)	**33.64 (2.7%)**	24.90 (24.0%)
25	38.07	45.88 (20.5%)	**36.40 (4.4%)**	27.46 (27.9%)
